# Serum Chemokines CCL3 and CCL7 as Complementary Diagnostic Biomarkers Across Tumor Grades in Clear Cell Renal Cell Carcinoma

**DOI:** 10.3390/ijms27052490

**Published:** 2026-03-08

**Authors:** Weronika Sokólska, Monika Zajkowska, Agnieszka Kulczyńska-Przybik, Tadeusz Werel, Karolina Orywal

**Affiliations:** 1 Department of Biochemical Diagnostics, Medical University of Bialystok, Waszyngtona 15A, 15-269 Bialystok, Poland; weronika.sokolska@sd.umb.edu.pl; 2 Department of Neurodegeneration Diagnostics, Medical University of Bialystok, Waszyngtona 15A, 15-269 Bialystok, Poland; monika.zajkowska@umb.edu.pl (M.Z.);; 3 Department of Urology, Medical University of Bialystok, M. Skłodowskiej-Curie 24A, 15-276 Bialystok, Poland

**Keywords:** clear cell renal cell carcinoma, chemokines, CCL3, CCL7, serum biomarkers, cancer diagnostics, immune signaling, renal cancer

## Abstract

The long asymptomatic period of clear cell renal cell carcinoma, which leads to delayed diagnosis and poorer prognosis, poses a global challenge. Chemokines play a pivotal role in immune regulation and tumor progression, making them promising biomarker candidates. This study aimed to evaluate the usefulness of the C-C motif chemokine ligand 3 (CCL3) and C-C motif chemokine ligand 7 (CCL7) by assessing their serum concentrations in 40 patients with stage G1 + G2 and stage G3 + G4 renal cancer, as well as in 58 healthy volunteers. Chemokine concentrations were measured using a multiplex Luminex assay and analyzed statistically, including receiver operating characteristic (ROC) analysis. Serum CCL3 concentrations were significantly elevated in ccRCC patients compared to controls and increased with tumor grade, with the highest levels observed in patients with advanced disease (G3+G4). In contrast, serum CCL7 levels were significantly lower in ccRCC patients than in healthy individuals, with no significant differences between tumor grade subgroups. ROC analysis revealed comparable diagnostic performance of CCL3 and CCL7, with CCL3 showing a slightly higher area under the curve. CCL3 showed high sensitivity, whereas CCL7 exhibited higher specificity than sensitivity, and a relatively high positive predictive value, consistent with its inverse regulation in ccRCC. These findings suggest that serum CCL3 and CCL7 are oppositely regulated in ccRCC and may serve as complementary non-invasive biomarkers for renal cancer detection.

## 1. Introduction

Kidney cancer is a disease with a steadily increasing incidence, estimated at 400,000 new cases annually, according to GLOBOCAN 2020 data. It ranks 9th in terms of malignancy incidence in men and 14th in women, as well as in the global population [1]. The disease most commonly affects older adults, with the mean age at diagnosis being approximately 75 years [2]. Renal cell carcinoma (RCC) is the most common, accounting for over 85% of patients. It ranks third among all urinary tract cancers, accounting for 3% of malignancies in adults and 80–90% of renal tumors [3].

Diagnosis is based on histological evaluation, including morphology and immunohistochemistry. The disease often develops asymptomatically for a prolonged period, resulting in diagnosis at more advanced stages and poorer prognosis. Standard treatment involves partial or radical nephrectomy [4]. Approximately 35% of patients with kidney cancer develop metastases, which spread primarily via the bloodstream and lymphatic system. The most common site of metastases is the lungs, accounting for approximately 70% of cases, but they can also involve lymph nodes, bones, and the liver. Because clear cell renal cell carcinoma (ccRCC) exhibits a high metastatic potential and metastases develop in approximately one-quarter of patients despite surgery, accurate assessment of recurrence risk is essential [5,6]. Therefore, it is crucial to identify and study the role of new molecules that can become diagnostic, prognostic, or predictive biomarkers [7].

**Figure 1:**
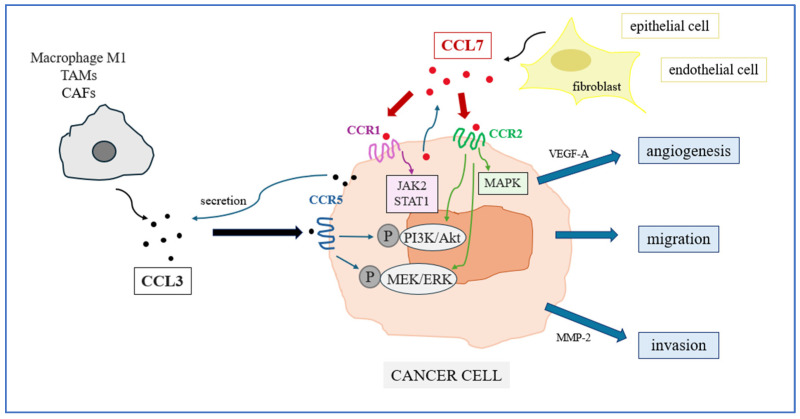
The role of chemokines CCL3 and CCL7 in signaling pathways leading to renal cancer progression.

Chemokines are a large group of proteins belonging to the cytokine family, with molecular masses ranging from 8 to 12 kDa. They are chemotactic factors that regulate intracellular signaling cascades, modulating the activity of immune cells in both physiological and pathological states [8]. Chemokines are involved in both antitumor and protumor immune responses and, through their ability to modulate the tumor microenvironment, represent attractive targets for investigation in many cancers, including renal cell carcinoma (Figure 1) [9].

Numerous studies, including those by Jianming Weng et al., confirm the close relationship between chemokines in the tumor microenvironment and immune infiltration and, consequently, prognosis in patients with renal cancer. They indicate significantly altered levels of CXC motif (CXC) chemokines and C-C motif (CC) chemokines, which are associated with the activation of numerous signaling pathways, participation in immune infiltration, and drug resistance [5]. Qu et al. found the usefulness of assessing the expression of CXC motif chemokine ligand 10 (CXCL10) in tumor tissues as a good candidate for a prognostic biomarker and a potential treatment target for renal clear cell carcinoma (KIRC) [6]. In turn, Esteban et al. report that elevated serum levels of this protein are associated with shortened Progression-Free Survival (PFS) and Overall Survival (OS) in a prospective cohort of patients with metastatic renal cell carcinoma [10].

The C-C motif chemokine ligand 3 (CCL3) and the C-C motif chemokine ligand 7 (CCL7) are chemokines from the CC chemokine family.

Both chemokines exert their biological effects by binding to CCR1, CCR2, and CCR5 receptors, which are widely expressed on various immune cells and tumor cells, suggesting that they modulate similar pathways of cell migration and immune signals in the tumor microenvironment [11]. Due to their shared origin, overlapping biological effects, involvement in similar signaling pathways, and established roles in inflammation and cancer-related immune responses, these chemokines represent an interesting subject for analysis. Therefore, this study aims to evaluate the potential of chemokines CCL3 and CCL7 as new serum biomarkers and their diagnostic usefulness in patients with renal cell carcinoma.

## 2. Results

**Figure 2:**
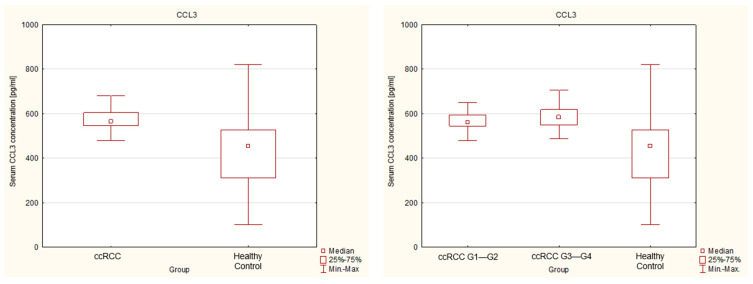
Serum concentrations of CCL3 in patients with clear cell renal cell carcinoma and healthy controls.

**Figure 3:**
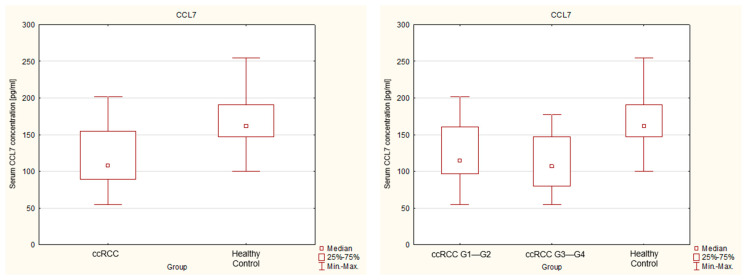
Serum concentrations of CCL7 in patients with clear cell renal cell carcinoma and healthy controls.

The concentrations of chemokines CCL3 and CCL7 in the serum of renal cancer patients and healthy patients (control group) are presented in Table 1. We demonstrated that both CCL3 and CCL7 are detectable in the serum of healthy individuals and patients with renal cancer. A comparative analysis revealed statistically significant differences in serum chemokine levels between renal cancer patients and the control group. The concentration of the chemokine CCL3 in renal cancer patients was significantly higher than in the control group. The median CCL3 concentration in the cancer group was 564.887 pg/mL, whereas in the control group it reached 454.835 pg/mL. Moreover, a progressive increase in CCL3 concentration was observed with increasing tumor grade. We found a higher median value of 585.197 pg/mL in the subgroup of patients with advanced cancer, classified as stage G3—G4, compared to the subgroup of patients with stage G1— G2 cancer, where it was 560.635 pg/mL. These results suggest a potential association between serum CCL3 levels and tumor aggressiveness. A nonparametric test (Mann–Whitney U) comparing the levels of the chemokine CCL7 showed that in the entire renal cancer study group, the concentration of this chemokine was statistically lower compared to the healthy control group (in all cases, *p* < 0.05). However, there was no statistical significance between the subgroup of patients with stage G1— G2 and the subgroup G3— G4. These findings indicate that, unlike CCL3, serum CCL7 levels are not associated with tumor grade. The distribution of serum CCL3 and CCL7 concentrations in the analyzed groups is illustrated in Figure 2 and Figure 3, respectively.

Box plots represent the median (central line), interquartile range (IQR) (box), and minimum–maximum values (whiskers). Individual data points represent single measurements. Statistical significance was assessed using the Mann–Whitney U test.

Box plots represent the median (central line), interquartile range (IQR) (box), and minimum–maximum values (whiskers). Individual data points represent single measurements. Statistical significance was assessed using the Mann–Whitney U test.

**Figure 4:**
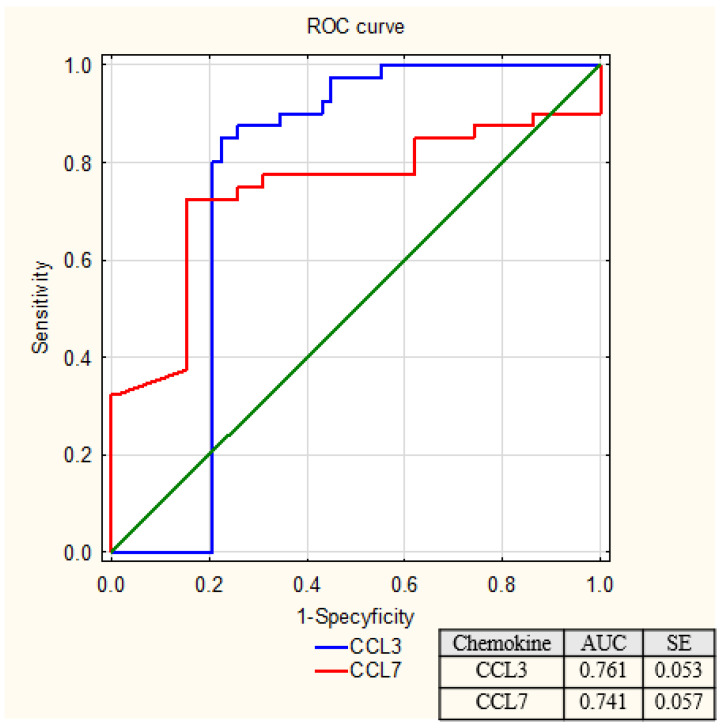
Receiver operating characteristic (ROC) curves for CCL3 and CCL7 in the diagnosis of clear cell renal cell carcinoma.

Table 2 presents the diagnostic utility parameters of the chemokines CCL3 and CCL7. Receiver operating characteristic (ROC) curve analysis was performed to evaluate the diagnostic performance of both chemokines (Figure 4).

The highest sensitivity (SE) of 85% was achieved by the chemokine CCL3. For the chemokine CCL7, the diagnostic sensitivity was 72.5%, and specificity was 84.5%. Positive predictive value (PPV) and negative predictive value (NPV) were calculated for both chemokines. For CCL3, the PPV was 72%, while for CCL7 it was 76.3%. The NPV for CCL3 was 88%, exceeding the NPV of CCL7 at 81.7%. These findings indicate that CCL3 demonstrates superior overall diagnostic performance. Combined analysis of CCL3 and CCL7 resulted in higher specificity, positive predictive value (PPV), and overall accuracy (ACC) compared to the individual chemokine measurements, indicating an improvement in diagnostic performance.

**Figure 5:**
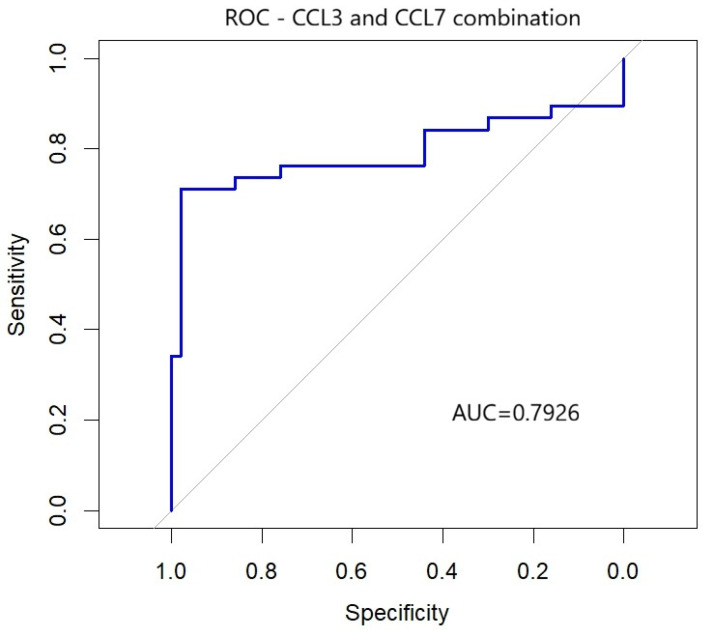
Receiver operating characteristic (ROC) curve for combined CCL3 and CCL7 analysis in the diagnosis of clear cell renal cell carcinoma.

Given that lower serum CCL7 concentrations were associated with ccRCC, ROC analysis for this chemokine was performed considering CCL7 as a destimulant. We observed that the area under the ROC curve (AUC) for CCL3 and CCL7 was similar, and was 0.761 and 0.741, respectively. The combined ROC analysis of CCL3 and CCL7 using a logistic regression-derived predictor demonstrated good discriminative ability between ccRCC patients and healthy controls, with an area under the curve (AUC) of 0.793 (Figure 5).

**Figure 6:**
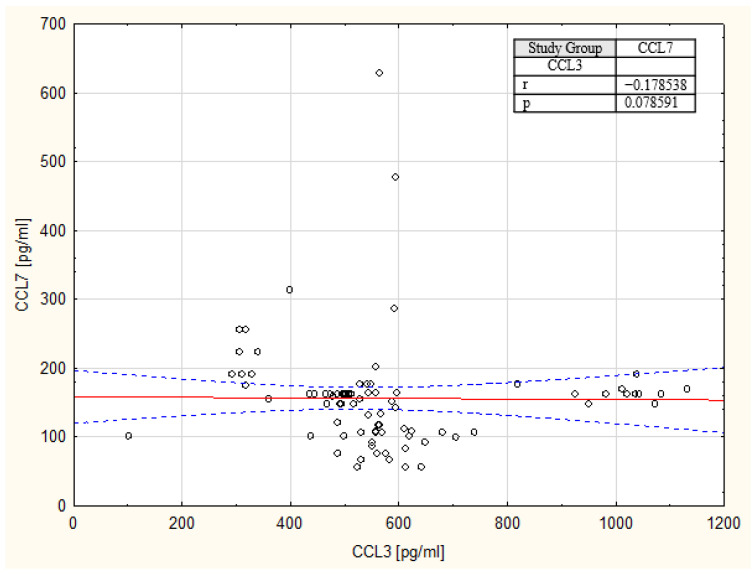
Spearman correlation between serum CCL3 and CCL7 concentrations in patients with clear cell renal cell carcinoma.

In order to assess the strength and direction of the monotonic relationship between variables, we used Spearman correlation and created a scatterplot of the CCL3 and CCL7 variables, presented in Figure 6. Correlation analysis revealed a weak relationship between serum CCL3 and CCL7 concentrations, suggesting that these chemokines may reflect distinct biological processes.

## 3. Discussion

Renal cancer is a heterogeneous disease characterized by a long asymptomatic period. Detection of renal lesions is most often incidental, during imaging studies such as computed tomography (CT), magnetic resonance imaging (MRI), or ultrasonography (USG) during diagnostic workup for other diseases [3]. By the time of detection, patients are often already in an advanced stage of cancer development, which translates into a poorer prognosis. The gold standard of treatment is radical or partial nephrectomy. Further patient follow-up is crucial to exclude potential disease progression and metastasis [12]. Currently, there are no effective biological biomarkers that address the issues of diagnosis, prognosis, and monitoring of patients with renal cell carcinoma. The search for new parameters that could fulfill these functions, especially in the initial stage when the first neoplastic lesions appear, is a global challenge for scientists. Growing evidence points to the crucial role of chemokines in signaling pathways involved in tumorigenesis. Due to the limited number of studies on the usefulness of selected CC chemokines in renal cancer, we decided to conduct this analysis focusing on CCL3 and CCL7, chemokines belonging to the CC subfamily and possessing two adjacent cysteines at the N-terminus [13,14].

The chemokine CCL3 is a cytokine in the chemokine family, also known as macrophage inflammatory protein-1α (MIP-1α). Its receptors are CCR1, CCR5, and CCR9. The main cells producing CCL3 include macrophages, monocytes, T cells, and B lymphocytes. It can induce the migration and infiltration of chemotactic immune cells, including dendritic cells, neutrophils, monocytes, macrophages, NK cells, and T cells, into target organs and inflammatory sites. It also controls the proper functioning of cytotoxic CD8+ T lymphocytes, which gives the chemokine CCL3 protective and antitumor effects. Modulating an effective immune response, it prevents mutated cells from escaping the body’s control. However, scientists have demonstrated its high expression in cancer cells and tumor-associated cells, TAMs, MDSCs, and MSCs, which translates into promoting tumor development [15,16]. Due to the pleiotropic nature of its occurrence and functions, it is present in the serum of both healthy and diseased individuals, both physiologically and pathologically, our analysis confirmed, with the key difference being its expression levels. In our study, we found that the concentration of the chemokine CCL3 in the serum of renal cancer patients was significantly higher compared to that of the healthy control group (*p* < 0.001). This can be explained by the fact that it is also secreted into the bloodstream by tumor cells and by elements of the tumor microenvironment (TME), hence its likely increased levels. We observed a correlation between tumor stage and CCL3 levels, as patients with later-stage disease (G3 and G4), which constituted one of our two study subgroups, had significantly higher CCL3 levels compared to patients with G1 and G2 disease, which is the initial stage, without necrotic lesions and without metastases to regional lymph nodes or distant metastases according to TNM classification criteria [17]. The literature indicates that the chemokine CCL3 recruits CAFs via the CCR5 receptor, thus contributing to progression and metastasis, which further explains its increase in patients with advanced renal cancer [15]. This raises the question of how CCL3 levels change in these patients in other material, namely, tumor tissue. Numerous studies have demonstrated changes in various chemokines in the tumor microenvironment, which results from immune infiltration. Jianminga Weng et al. found significantly increased levels of the chemokine CCL3 mRNA, with a fold change of >1 in tumor tissue compared to healthy kidney tissue [18]. Daniela Vargová et al. obtained similar results, finding elevated CCL3 levels in renal cell carcinoma, which may indicate the potentially pro-tumor nature of this chemokine [19]. In addition to serum and tumor tissue fragments, urine is a biological material in which new diagnostic biomarkers can be sought. In another study, Daniela Vargová et al. determined CCL3 concentrations in both plasma and urine. They demonstrated that the urine of patients with clear cell renal cell carcinoma had significantly higher CCL3 concentrations both before and after surgery compared to control urine, while the same determination in plasma did not demonstrate statistical significance. No statistically significant correlation was found between plasma and preoperative urine samples from patients with renal cancer [20]. Sylwia Popek-Marciniec et al. in their study also demonstrated significantly higher CCL3 concentrations in the serum of patients from the study group compared to the control group; however, the study group consisted of patients diagnosed with chronic lymphocytic leukemia [21]. In turn, a study by Marjorie De la Fuente Lópezi et al., focusing on the relationship between the chemokines CCL2, CCL3, and CCL4 and the tumor microenvironment in colorectal cancer, showed that CCL3 was the only chemokine whose level in the plasma of patients with colorectal cancer was significantly higher compared to the control group [22].

The chemokine CCL7, also known as monocyte chemotactic protein 3, is expressed primarily in fibroblasts, monocytes, and tumor cells [13,23]. For the first time, CCL7 was characterized from the supernatant of human osteosarcoma and has therefore become a subject of research and exploration of its role in the progression of other cancers. It exerts its functions, such as monocyte recruitment and induction of calcium ion influx, through binding to the CCR1, CCR2, and CCR3 receptors [24]. Our study demonstrated that the highest mean levels of the chemokine CCL7 were present in the serum of healthy controls. We observed statistically lower concentrations of this parameter in the serum of renal cancer patients in both the first subgroup (stage G1—G2) and the second (stage G3—G4) compared to healthy volunteers. However, we did not observe statistically significant differences in CCL7 concentrations between the two subgroups studied. The observed decrease in serum CCL7 levels in ccRCC patients may reflect complex regulation of the chemokine axis, including increased binding and consumption of CCL7 by CCR receptors within the tumor microenvironment [25]. Furthermore, it may be due to sequestration of CCL7 in tumor tissue or increased urinary excretion due to renal involution. It is possible that ccRCC cancer cells may directly inhibit CCL7 production [26,27].

We searched available sources, but unfortunately, we found no studies that would confirm or refute our findings regarding the chemokine CCL7 in the serum of patients with renal cancer. In their study, Hikari Chidimatsu et al. examined CCL7 levels in the serum of patients with metastatic colorectal cancer (mCRC) before treatment. They found that significantly high CCL7 levels observed in the study group were associated with poorer treatment outcome, prognosis, and overall survival compared to patients with lower levels of this chemokine, suggesting its usefulness as a prognostic factor for mCRC [28]. Miran Jeong et al., in turn, examined the expression level of CCL7 in macrophages stimulated with ovarian cancer compared to macrophages from healthy controls. Obtaining significantly higher values in the study group, they suggested its role in ovarian cancer cell invasion and migration via activation of the CCL7-CCR3 axis via the ERK pathway [29]. To date, most studies of CCL7 chemokine levels have been based on its assessment in tumor tissue, including non-small cell lung cancer [30], colorectal metastases [31], and oral squamous cell carcinoma [32], relative to healthy tissue. Its high expression in many tumors suggests an additional role in disease progression.

Based on our study, we found a sensitivity of 85% for the serum chemokine CCL3 and a specificity of 77.6%, suggesting its diagnostic potential in screening for renal cancer. In contrast, the chemokine CCL7 achieved high diagnostic specificity (84.5%) and a relatively high positive predictive value, but lower sensitivity compared to CCL3, suggesting its usefulness as a complementary marker, particularly when interpreted as a destimulant in ROC analysis. Similar approaches have been reported in other cancers, supporting the use of chemokines as diagnostic biomarkers. Plasma CCL3 concentration in nasopharyngeal carcinoma showed high diagnostic accuracy (AUC ~0.91, sensitivity 90%, specificity >80%), particularly when combined with other markers [33]. In necrotizing enterocolitis, serum CCL3 also exhibited high sensitivity (~83%) and NPV (~80%), with performance further improved in multi-marker panels [34]. These studies indicate that chemokines can serve as valuable components of multi-parametric biomarker panels, enhancing the detection of disease. Our findings highlight the complementary nature of these chemokines, with CCL3 providing high sensitivity and CCL7 providing high specificity, supporting the combined assessment of these parameters for improved diagnostic accuracy. It should be emphasized that chemokines, including CCL3 and CCL7, are pleiotropic mediators involved in multiple immune and inflammatory pathways beyond carcinogenesis itself. Their serum concentrations do not solely reflect tumor activity, but may be modulated by systemic inflammation of various etiologies, comorbidities such as diabetes, hypertension, infections, and the effects of pharmacotherapy, including immunomodulatory, anti-inflammatory, and targeted therapies [35,36,37]. Consequently, the interpretation of their potential diagnostic or prognostic value in renal cancer requires caution and consideration of possible confounding factors that may lead to an inadequate assessment of their actual relationship with the neoplastic process.

### Limitations and Future Perspectives

Despite several important findings, this study has limitations that should be acknowledged. The relatively small sample size, particularly within tumor grade subgroups (G1—G2 vs. G3—G4), may limit statistical power and restrict the ability to detect subtle differences between clinical stages. Moreover, the single-center design may reduce the generalizability of the results to broader populations. Therefore, the findings should be interpreted with caution and considered exploratory. Although the control group consisted of healthy volunteers without a history of malignancy, autoimmune disorders, chronic inflammatory diseases, or current infection, circulating chemokine levels may be influenced by other unmeasured biological or environmental factors. Residual confounding cannot be entirely excluded. Additionally, the analysis was based exclusively on serum measurements of CCL3 and CCL7. The absence of tissue-level validation (e.g., immunohistochemistry or gene expression analysis in tumor specimens) limits the ability to directly correlate circulating chemokine levels with tumor microenvironment activity. Integrating serum and tissue analyses in future studies would provide a more comprehensive understanding of the biological role of these chemokines in ccRCC progression. Finally, larger, multicenter studies are necessary to validate the observed associations and to determine the potential clinical utility of CCL3 and CCL7 as diagnostic or prognostic biomarkers.

## 4. Materials and Methods

The study included 40 patients (28 men and 12 women; mean age 58 years, range 34–83 years) diagnosed with clear cell renal cell carcinoma, who underwent surgical treatment at the Department of Urology, University Clinical Hospital in Białystok. The patients were divided into two groups: 24 patients in stages G1 and G2, and 16 patients in stages G3 and G4. Exclusion criteria included: prior oncological treatment, active infection, autoimmune disease, chronic inflammatory disorders, and treatment with immunomodulatory drugs. The majority of patients presented with non-metastatic disease at diagnosis (*n* = 38), while metastatic disease (M1) was observed in two cases. The control group consisted of 58 healthy volunteers (30 men and 28 women; mean age 58 years, range 51–67 years) without a history of malignancy, autoimmune disorders, chronic inflammatory diseases, or current infection. The study material consisted of serum samples collected before treatment and before initiation of any therapeutic interventions to minimize potential effects of medications on chemokine levels. Additionally, participants reported any regular or chronic use of medications that could influence immune parameters. Peripheral blood was collected from each participant in a tube without an anticoagulant. After clot formation, the material was centrifuged to isolate the serum, separated, and stored at −80 °C until analysis. The chemokines studied were assayed and measured using a Luminex 200 analyzer and Luminex Human Discovery assay plates, supplied by R&D Systems, Abingdon, UK. This method allows for the simultaneous determination of multiple parameters using a multiplexing, multiparametric, fluorescent microsphere laser reading system. Each standard, control, and sample was assayed in duplicate according to the manufacturer’s protocols. Analysis of the samples’ concentrations (based on mean fluorescence intensity) was performed by the xPONENT 3.1.971.0 quantification software (Luminex, Austin, TX, USA). The clinical diagnosis of patients was established based on histopathological examination of material obtained during surgical resection of the renal tumor. Characteristics of the control and study groups are presented in Table 3.

The research protocol was approved by the Medical University of Białystok’s Human Care Committee located in Białystok, Poland (Approval Nr APK.002.482.2025). All patients from the tested and control groups gave their informed consent for the examination.

The statistical computer program STATISTICA 13 was used for statistical analysis of the results. Normality of distribution was assessed using the Shapiro–Wilk test. Because the distribution of variables differed from normal after the initial analysis, the nonparametric Mann–Whitney U test was used to compare two independent samples, and the Kruskal–Wallis test was used. Data are presented as median, minimum, and maximum range. Diagnostic sensitivity, specificity, and positive and negative predictive values of the test results (SE, SP, PPV, and NPV, respectively), which determine the diagnostic usefulness of the measured parameters, were calculated using cutoff values calculated using the Youden index. A receiver operating characteristic (ROC) curve was defined to estimate the diagnostic accuracy of all tested parameters. To compare the data obtained across groups, a Spearman rank correlation test was performed, and a scatterplot was prepared. Results were considered statistically significant at a significance level of *p* < 0.05. For the combined analysis of CCL3 and CCL7, logistic regression, ROC analysis, and calculation of diagnostic performance metrics (sensitivity, specificity, accuracy, PPV, and NPV) were performed using RStudio (R version 4.5.2) with the pROC package.

## 5. Conclusions

In summary, CCL3 and CCL7 are detectable in the serum of healthy individuals and patients with renal cancer. CCL3 levels increase with tumor stage, whereas CCL7 decreases in patients compared to controls. Combined assessment of both chemokines may improve early detection of renal cancer. Further studies are required to confirm their diagnostic utility and clinical applicability.

## Figures and Tables

**Table 1:** Serum concentrations of chemokines CCL3 and CCL7 in cancer patients and controls.

Group	Mean	Median	Min.	Max.	SD
CCL3					
ccRCC Group	572.95	564.89	399.87	739.00	61.60
ccRCC G1— G2	561.24	560.64	399.87	680.80	57.73
ccRCC G3—G4	590.50	585.20	488.59	739.00	64.80
ccRCC female	549.73	550.68	399.87	649.22	60.97
ccRCC male	540.58	339.57	101.00	1131.23	403.22
Control Group	491.64	454.84	101.00	1131.23	298.40
Control female	584.89	568.01	101.00	1131.23	303.99
Control male	540.58	339.57	101.00	1131.23	403.22
p^a^ < 0.001 p^b^ < 0.002 p^c^ < 0.001 p^d^ = 0 p^e^ < 0.001 p^f^ = 0.207					
CCL7					
ccRCC Group	142.28	108.08	55.19	628.00	110.80
ccRCC G1— G2	144.50	114.17	55.19	476.90	94.14
ccRCC G3—G4	138.90	106.70	55.19	628.00	135.40
ccRCC female	126.19	101.00	55.18	312.36	76.79
ccRCC male	151.93	116.43	55.19	627.54	127.50
Control Group	166.17	161.97	100.19	254.89	39.92
Control female	151.51	138.08	55.18	627.54	93.90
Control male	159.23	161.69	101.00	254.89	44.76
p^a^ < 0.001 p^b^ < 0.009 p^c^ < 0.001 p^d^ = 1.0 p^e^ < 0.05 p^f^ < 0.05					

SD: Standard deviation; Statistically significant differences were defined as comparisons resulting in *p* < 0.05. Data are expressed as pg/mL; p^a^—cancer patients vs. controls; p^b^—G1— G2 group vs. controls; p^c^—G3—G4 group vs. controls; p^d^—G1—G2 group vs. G3—G4 group; p^e^—ccRCC female vs. control female; p^f^—ccRCC male vs. control male.

**Table 2:** Diagnostic performance parameters of serum CCL3 and CCL7 in clear cell renal cell carcinoma.

	Cut-Off	Diagnostic Sensitivity (%)	Diagnostic Specificity (%)	Positive Predictive Value (%)	Negative Predictive Value (%)	Accuracy (%)
CCL3	531.07	85.0	77.6	72.0	88.0	80.6
CCL7	143.18	72.5	84.5	76.3	81.7	79.6
CCL3+CCL7	0.457	71.1	98.0	96.4	81.7	86.4

Cut-off values were determined using the Youden index to maximize the sum of sensitivity and specificity. CCL7 was analyzed as a destimulant, as lower serum concentrations were associated with ccRCC.

**Table 3:** Clinical characteristics of the control group and patients with clear cell renal cell carcinoma (ccRCC).

Characteristic	Control Group (n = 58)	ccRCC Patients (n = 40)
Sex, *n* (%)		
Male	30 (51.7)	28 (70.0)
Female	28 (48.3)	12 (30.0)
Tumor grade, n (%)		
G1—G2	—	24 (60.0)
G3—G4	—	16 (40.0)
Metastatic status (M stage), n (%)		
M0	—	38 (95.0)
M1	—	2 (5.0)
Sex distribution by grade, n		
G1—G2 (Male/Female)	—	17/7
G3—G4 (Male/Female)	—	11/5

## Data Availability

The original contributions presented in this study are included in the article. Further inquiries can be directed to the corresponding author.
